# Myositis ossificans of the thigh causing external compression of the superficial femoral artery and vein: A case report

**DOI:** 10.1097/MD.0000000000022810

**Published:** 2020-10-23

**Authors:** Sung Il Wang, Eun Hae Park, Hong Pil Hwang, Jung Ryul Kim

**Affiliations:** a Department of Orthopaedics Surgery; b Department of Radiology; c Department of Surgery, Jeonbuk National University Medical School, Research Insitute of Clinical Medicine of Jeonbuk National University–Biomedical Research Insitute of Jeonbuk National University Hospital, 567 Baekje-ro, Dukjin-gu, Jeonju 561-756, Republic of Korea.

**Keywords:** arterial reconstruction, femoral vessel, myositis ossificans, obstruction

## Abstract

**Rationale:**

Myositis ossificans (MO) is a benign condition characterized by heterotopic bone formation in the skeletal muscle of extremities. Marked variation can occur in the incidence and location of the bone formed as well as resulting complications. Femoral vessel obstruction caused by MO is an extremely rare but disabling complication. Arterial occlusion may aggravate ischemic conditions, resulting in necrosis in the lower extremity.

**Patient concerns:**

We report a 41-year-old female with progressive pain and swelling of the right thigh region for 1 year.

**Diagnoses:**

We diagnosed it as obstruction of the superficial femoral artery and vein caused by external compression of the MO between the sartorius and vastus medialis of the thigh.

**Interventions and outcomes:**

Adherent tissues and mass were excised with care without damaging the femoral artery or the vein. However, normal morphology did not recover due to loss of elasticity of femoral vessels. Therefore, after resection of the narrowed region of the femoral artery, a femoral-to-femoral graft interposition using the greater saphenous vein was performed. At 12 months after the surgery, vessel reconstruction computed tomography images confirmed normal continuous flow of the femoral artery.

**Lessons:**

Vascular compression and peripheral inflammatory response due to MO can cause loss of normal vascular morphology. Surgical excision of the mass and the involved femoral artery segment followed by femoral arterial reconstruction should be considered for lesions that do not spontaneously regress to prevent functional impairment and secondary complications in extremities.

## 1 Introduction

Myositis ossificans (MO) is a benign condition characterized by heterotopic bone formation in skeletal muscle of extremities.^[1]^ It is a well-recognized but poorly understood disease caused by inflammation and subsequent ossification of the muscle and other soft tissues that normally exhibit no ossification properties.^[2]^ It is often confused with other soft tissue tumors, as these conditions commonly show inflammatory phenomena. Although MO treatment is usually conservative, its incidence, location of the bone formed, and resulting complications can show marked variations. Reported complications include peripheral nerve entrapment, pressure ulcers, and functional impairment due to ankyloses.^[3,4]^ However, vessel occlusion associated with MO is extremely rare. To the best of our knowledge, only 3 cases of MO resulting in vessel obstruction have been reported.^[5–7]^ Here, we report a case of MO in a 41-year-old female without underlying diseases. The MO caused painful swelling of the thigh through extrinsic compression of the superficial femoral artery and vein. Painful swelling and limited motion of the knee joint persisted without spontaneous regression for 1 year. Therefore, surgical excision of the mass and the involved femoral artery segment were performed followed by femoral arterial reconstruction using the greater saphenous vein.

## 2 Consent

The patient signed informed consent for publishing this case report and any accompanying image. The ethical approval of this study was waived by the ethics committee of Jeonbuk National University Hospital because this study was a case report and the number of patients was less than 3.

## 3 Case report

A 41-year-old female patient was referred to our clinic because of progressive pain and swelling of the right thigh region for 1 year. She often had repetitive blunt microtrauma to the medial aspect of the right thigh against corners of tables while serving at a restaurant. In the general surgery department, she was diagnosed as having muscle inflammation a year ago. She was treated with physical therapy and medication. However, there was no improvement of the symptom. Physical examination at our clinic revealed that the right thigh circumference was 2 cm larger than that of the left. There was tenderness along the medial third of the thigh along with pain upon passive stretching. The patient had no acute inflammatory signs. Although the branching of the toe was present, the pulsation of dorsalis pedis artery seemed to decrease slightly compared with the contralateral side.

**Figure 1:**
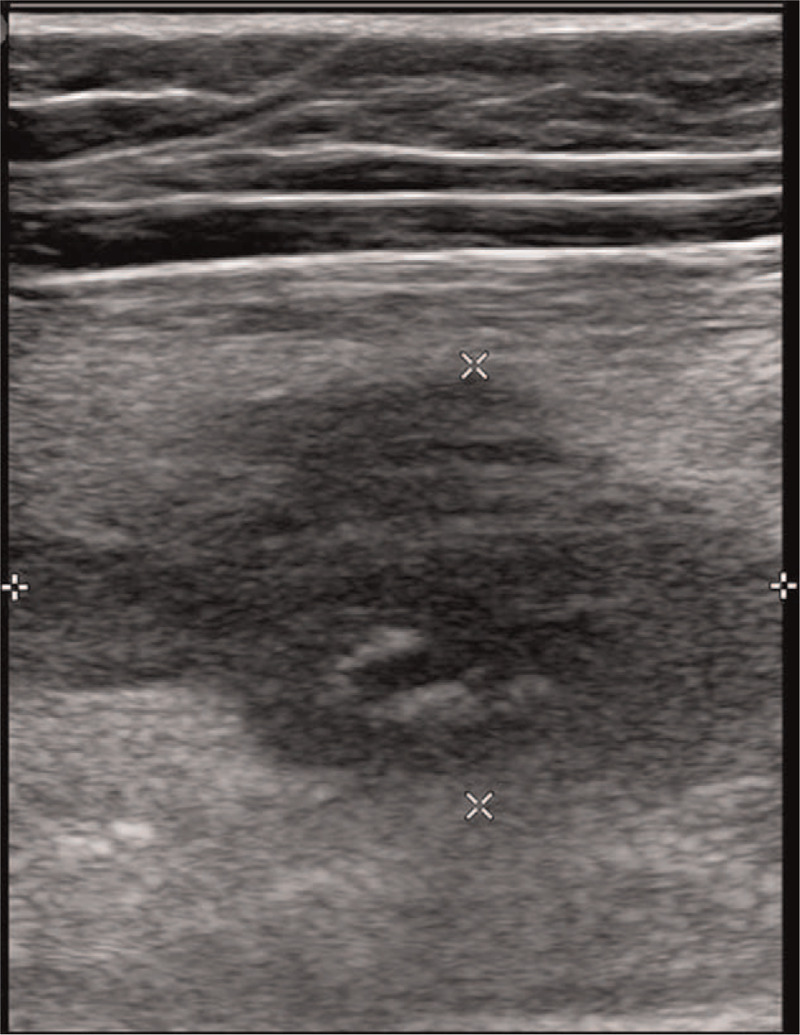
Musculoskeletal ultrasound of a 41-year-old female with right lower extremity pain and swelling. At the medial aspect of the thigh, there was an ill-defined hypoechoic mass-like lesion with peripheral calcification.

**Figure 2:**
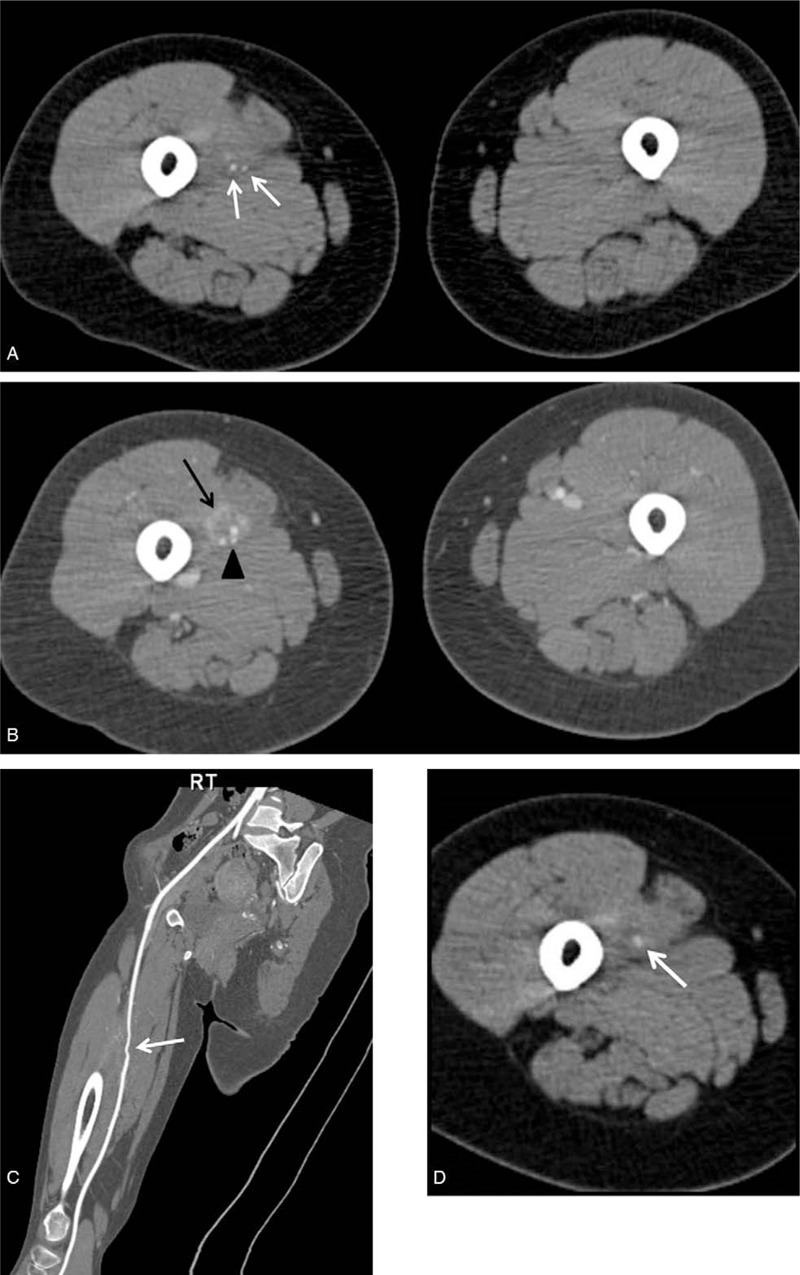
Computed tomography (CT) images obtained at the general surgery clinic 1 year before referral and at 12-month follow up. (A) Before contrast enhancement, focal calcification (white arrows) was observed in the medial aspect of the right thigh. (B) Following contrast enhancement, obliteration of the vein was observed with replacement by the mass, which showed peripheral enhancement (black arrow). Calcification was located in the center of the lesion (black arrow head). The diameter of the artery was reduced compared to that of the left side. (C) Focal narrowing of the artery was verified in vessel reconstruction images. (D) CT imaging at 12 months follow-up showing absence of calcification that was observed previously while new focal calcification could be seen.

**Figure 3:**
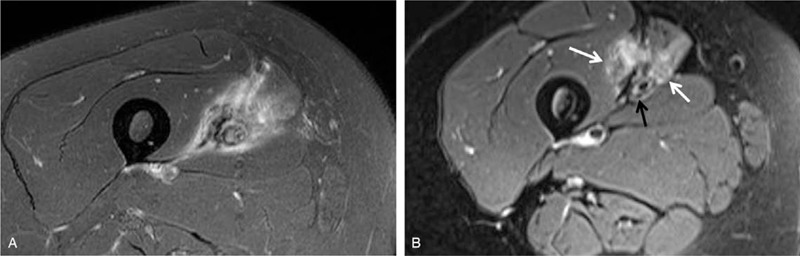
Magnetic resonance (MR) imaging performed at the general surgery clinic 1 year before referral and at 12-month follow up. (A) Narrowing of the artery could be seen in T2-weighted image. The lesion showed an ill-defined heterogeneous signal and severe perilesional edema. (B) MRI T2-weighted imaging at 12 months still showed an ill-defined lesion while the edema was reduced (white arrows). It was not possible to clearly differentiate the vein due to vessel collapse (black arrow).

**Figure 4:**
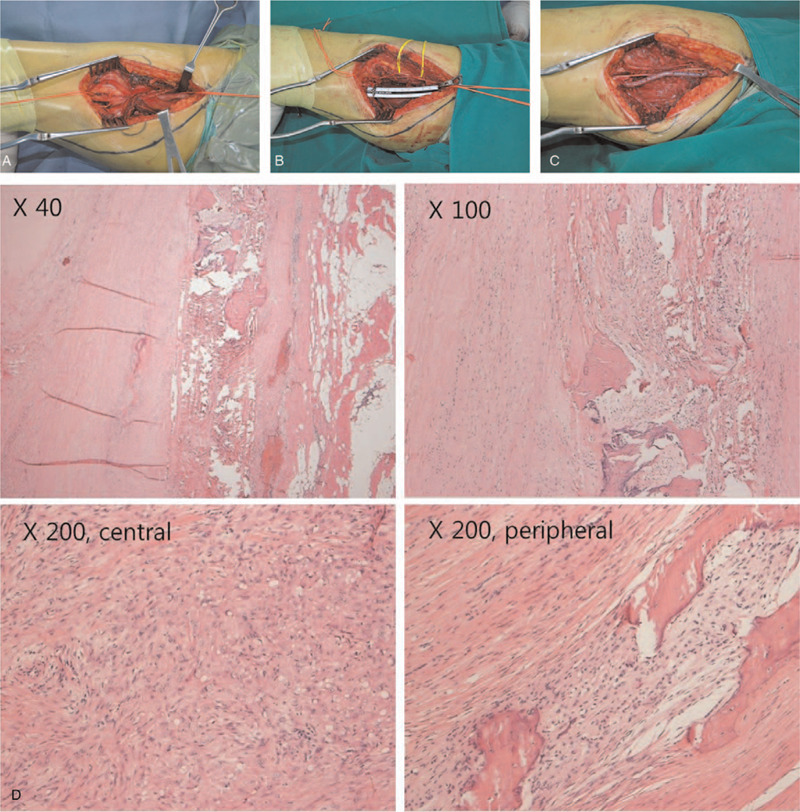
Surgical and histologic findings of the mass line lesion of the right thigh. (A) Intraoperative findings showed a hard, irregular margin mass-like lesion surrounding the middle portion of the femoral artery in the right proximal thigh. The mass was strongly adherent to adjacent muscles and femoral vessels. (B) Adherent tissues and mass were excised with care to avoid damaging the femoral artery. However, normal morphology did not recover due to loss of elasticity in the femoral artery. Therefore, resection of the narrowed region was performed with a size of 9 cm. (C) A femoral-to-femoral graft interposition using the greater saphenous vein was performed. (D) Hematoxylin and eosin-stained sections showing chronic inflammation with extensive fibrosis and ossification.

**Figure 5:**
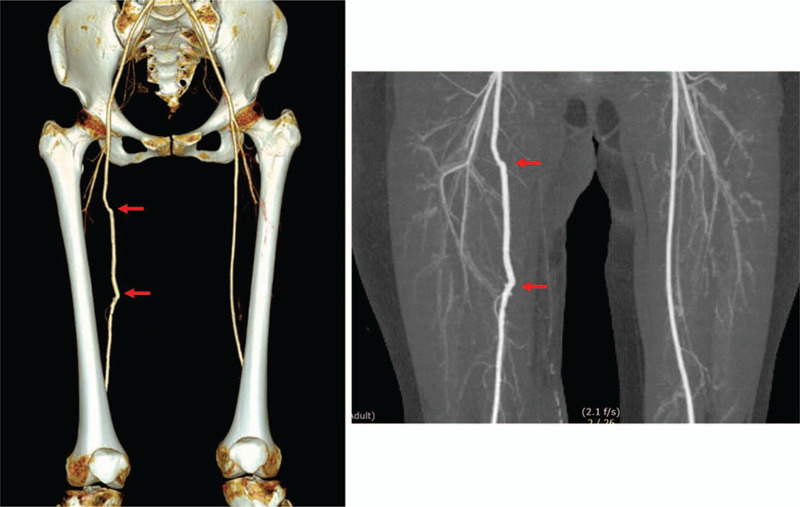
CT obtained at 12 months after excision of the mass and femoral-to-femoral graft interposition using the greater saphenous vein. Vessel reconstruction CT images confirmed normal continuous flow of the femoral artery.

Laboratory examination results were all within normal limits. Radiographs of the right femur showed no abnormal lesion either. Ultrasound examination revealed a 3.2 mm × 2.0 mm × 2.0 mm space occupying the lesion at the medial region of the thigh between the sartorius and vastus medialis (Fig. 1). Contrast-enhanced spiral computed tomography (CT) performed 1 year earlier showed a tumor-like lesion with ill-defined margins and a few tiny peripheral calcifications. The tumor showed peripheral enhancement. The right femoral vein was obliterated and the lesion encased the superficial femoral artery (Fig. 2A, B. Focal narrowing of the artery was verified by vessel reconstruction imaging (Fig. 2C). CT images performed at the time of referral to our clinic showed absence of calcification that was observed previously. However, new focal calcification was observed (Fig. 2D). Newly developed collateral vein was observed around the previously compressed femoral vein. Magnetic resonance (MR) imaging performed 1 year earlier revealed an ill-defined mass-like lesion that compressed the superficial femoral artery and vein. The lesion showed iso-intensity on T1-weighted imaging and heterogeneous high signal intensity on T2-weighted imaging accompanied by severe perilesional edema (Fig. 3A). A few focal peripheral calcifications were revealed as low signal foci on both T1- and T2-weighted images. MR images performed at the time of referral to our clinic showed progression to vessel collapse (Fig. 3B). The painful swelling and limited motion of the knee joint persisted without spontaneous regression for one year. Therefore, surgical excision of the mass was performed. The mass-like lesion surrounding the middle portion of the femoral artery and vein in the right proximal thigh had a hard, irregular margin. It was strongly adhesive to adjacent muscles and femoral vessels. Thus, these adherent tissues and mass with dimension of 5.0 x 5.0 x 2.5 cm were excised with care without damaging femoral vessels. However, normal morphology did not recover due to loss of elasticity of femoral vessels. Although both the femoral artery and vein lost normal vascular function, the collateral vein was well developed around the lesion. Thus, we performed a femoral-to-femoral graft interposition using the great saphenous vein after resection of only the narrowed region of the femoral artery (Fig. 4A--C). The examination performed immediately after surgery showed no difference in toe branching or pulsation of dorsalis pedis artery compared with the contralateral side. Histologically, the mass was diagnosed as MO with chronic inflammation and extensive fibrosis as well as ossification (Fig. 4D). Following the surgery, warfarin was prescribed during wound healing. The patient experienced no pain or swelling in the right thigh. At 12 months after the surgery, vessel reconstruction CT images confirmed normal continuous flow of the femoral artery (Fig. 5).

## 4 Discussion

Because of the self-limiting and benign nature of MO, its treatment in most cases is conservative. However, its incidence and location of the bone formed can show marked variations. Reported complications include peripheral nerve entrapment, pressure ulcers, and functional impairment due to ankyloses.^[3,4]^ However, vessel occlusion associated with MO is extremely rare. To the best of our knowledge, only 3 cases of MO resulting in obstruction of femoral vessels have been reported.^[5–7]^ One of these cases presented with MO that occluded the left common femoral artery.^[5]^ Arterial pulsation was restored by detaching the area with MO from the femoral artery. Another case consisted of extrinsic compression of the right femoral vein by the MO.^[6]^ Medical treatment was instituted with oral etidronate disodium. In another case, the MO compressed and obstructed the left common iliac vein.^[7]^ It was successfully treated with venous stenting and percutaneous angioplasty. Vascular compression and occlusion are known to cause ipsilateral lower extremity pain, swelling, and occasional vessel ulceration. If these symptoms persist, the normal pumping action of the muscle is lost, further exacerbating venous stasis and dependent edema. Meanwhile, arterial occlusion may aggravate ischemic conditions, resulting in necrosis in the lower extremity. In the present case, the occlusion of femoral artery and vein caused by external compression of MO in the thigh persisted without evidence of spontaneous regression after 1 year.

The mass was excised together with surrounding adherent tissues. However, the femoral artery did not return to normal morphology due to loss of elasticity. Therefore, a femoral-to-femoral graft interposition was performed using the greater saphenous vein.

In cases of acute or chronic painful inflammation of the lower extremity, it is essential to rule out deep vein thrombosis. In current case, there was no acute inflammation signs in the preoperative examination. The obstruction of superficial femoral artery and vein was caused by external compression of the mass-like lesion between the sartorius and vastus medialis of the thigh. Thus, deep vein thrombosis could be excluded.

MO is often misdiagnosed as tumors of some other soft tissues since these conditions commonly show inflammatory phenomena. In the present case, differential diagnosis based on clinical and radiographic data included the possibility of proliferative myositis and soft tissue sarcoma. Proliferative myositis is a rare benign tumor that develops in the skeletal muscle. Muscle fiber without discontinuation is a characteristic MRI finding of proliferative myositis.^[9]^ Other characteristic findings on histology include a “checkerboard’ pattern of myofibroblasts infiltrating surrounding healthy muscles and ganglion-like basophilic giant cells without any sign of atypical mitosis. In the current case, because the margin of the mass-like lesion was ill-defined and discontinuation of adjacent muscle fibers was observed with edema and fibrotic changes on MR imaging, proliferative myositis could be excluded.

Soft-tissue sarcomas are rare malignant tumors found in adult patients. Approximately 20% to 30% of soft tissue sarcomas contain calcification in the sarcomatous tissue. MRI shows a well-defined mass with intermediate signal intensity on T1 and high signal intensity on T2 sequences with heterogeneous contrast enhancement.^[10]^ They usually show an increase in the size of the mass and edema of adjacent soft tissues at follow-up MRI. However, in the present case, the patient's MRI at follow-up showed a decrease in the size of the mass-like lesion and edema of adjacent soft tissue. There were also changes in the calcification position of the lesion. Because these radiological findings are not common characteristics of soft-tissue sarcoma, soft-tissue sarcoma could be excluded. Histologic evaluation of the biopsy specimen showed no tumor either.

In conclusion, vascular compression and peripheral inflammatory response due to MO can cause loss of normal vascular morphology. Surgical excision of the mass and the involved femoral artery segment followed by femoral arterial reconstruction should be considered for lesions that do not spontaneously regress to prevent functional impairment and secondary complications in extremities.

## Author contributions

**Conceptualization:** Sung Il Wang, Jung Ryul Kim.

**Formal analysis:** Sung Il Wang, Eun Hae Park.

**Investigation:** Hong Pil Hwang.

**Project administration:** Hong Pil Hwang.

**Supervision:** Sung Il Wang, Jung Ryul Kim.

**Writing – original draft:** Sung Il Wang, Eun Hae Park.

**Writing – review & editing:** Jung Ryul Kim.
